# Fabrication of Self-Lubricating Porous UHMWPE with Excellent Mechanical Properties and Friction Performance via Rotary Sintering

**DOI:** 10.3390/polym12061335

**Published:** 2020-06-12

**Authors:** Xianwu Cao, Yuping Li, Guangjian He

**Affiliations:** National Engineering Research Center of Novel Equipment for Polymer Processing, Key Laboratory of Polymer Processing Engineering (SCUT), Ministry of Education, Guangdong Provincial Key Laboratory of Technique and Equipment for Macromolecular Advanced Manufacturing, South China University of Technology, Guangzhou 510641, China; LI_YUPING666@163.com (Y.L.); hegj@scut.edu.cn (G.H.)

**Keywords:** rotary sintering, ultra-high molecular weight polyethylene (UHMWPE), porous materials, self-lubricating

## Abstract

Porous ultra-high-molecular-weight polyethylene (UHMWPE) self-lubricating materials were designed and fabricated by a rotary sintering method, and the microstructure and properties were evaluated. Results showed that the rotary molding could not only significantly improve the molding efficiency but also formed uniform internal microstructures with high porosity, excellent mechanical properties, and low friction coefficient. Under oil lubricating conditions, the friction curve of samples quickly reached a steady state, the friction coefficient was reduced by 50%, and the repeat utilization was up to 99%. The following optimum sintering conditions were shown: Sintering temperature of 180 °C or 190 °C, sintering time determined as 10 min, and loading capacity of between 3.6 g and 3.8 g. Therefore, it is expected that this work will open a convenient and compatible strategy for fabricating porous materials with good self-lubricating performance.

## 1. Introduction

With the wide application of intelligent robots, drones, etc., the requirements for rapid conversion and motion transformation have been increased, and with reliability and tribological performance requirements of the corresponding friction pairs, joint connections have been gradually improved [1,2]. Aiming at the problems of friction and lubrication under special conditions, porous self-lubricating materials are attracting more and more attention due to their unique advantages such as good mechanical properties and excellent self-lubrication. To improve the friction properties, most porous materials have solid lubricants such as graphite [3], PTFE [4,5], and boron nitride (BN) [6]. However, under special conditions, the tribological properties of these materials are reduced and the service life is shortened. Therefore, porous polymer self-lubricating materials have emerged. They can realize the storage and transport of grease through the inter-connected hole structure inside the material. The use of porous self-lubricating materials in robot joints, drones, etc., can achieve a recirculating oil supply without the need for additional lubricating oil, achieving continuous lubrication and avoiding lubricant contamination. Some materials laden with lubricating oil are used for self-lubrication such as organogels [7], polyurethane [8], and diatomite [9]. The porous structure of these materials has a large specific surface area and a strong oil absorption capacity, which can achieve a good oil storage effect. However, they are not conducive to the preparation and application of mechanical parts, because of their poor mechanical properties.

In the manufacture of machine parts, the materials with high strength, low friction coefficient, and good chemical stability, such as polyimide (PI) [10,11,12], polyether ether ketone (PEEK) [13,14,15], and ultra-high-molecular-weight polyethylene (UHMWPE), have been widely utilized as porous materials. However, the high price of PI and PEEK limits their large-scale production/application [16,17,18]. Compared to other polymer porous materials, UHMWPE porous material products have the advantages of light weight, high specific strength, and good permeability [19,20,21]. Nowadays, research on the UHMWPE porous self-lubricating material is rare. Xiong et al. [22] improved the wear resistance of UHMWPE by filling carbon fibers in UHMWPE materials. Wu et al. [23] used NaCl as a pore-forming agent to prepare UHMWPE porous self-lubricating materials, and the wear of the material was reduced by 43%. Wang et al. [24] prepared a composite coating combined with a porous TiO_2_ layer, UHMWPE, and DMMPPS, achieving super-lubrication on the surface of the Ti6Al4V alloy. Chen et al. [25] cross-linked the PVA/ HA composite hydrogel on the surface of UHMWPE and obtained artificial articular cartilage with a low friction coefficient. However, most of them mainly focus on how to improve the wear resistance of the material.

Due to the high viscosity and poor fluidity of UHMWPE, conventional mechanical processing methods are difficult to process, and they are usually formed by special methods, such as thermally induced phase separation (TIPS) [26,27,28], thermally induced phase separation stretching (TIPS-S) [29], melt extrusion stretching [30,31], the nuclear track method [32], and powder sintering method [33,34,35]. Nevertheless, these methods have their deficiencies such as complicated operations, long molding times, and less choice of shape and size of the products. Moreover, due to the poor thermal conductivity of UHMWPE, uneven heating often occurs, which affects the subsequent use of porous products. Therefore, it is necessary to explore a new simple and efficient molding method to improve the heat conduction efficiency and optimize the microstructure of porous materials.

This investigation proposed a novel rotary sintering method based on the conventional sintering method to self-lubricating porous UHMWPE materials. A centrifugal force was applied to the material by the rotation of the mold to achieve the effect of pre-pressing. The influence of the different process parameters on the porous structure and mechanical properties of UHMWPE porous materials, as well as the tribological properties, was investigated in depth. This study verified the versatility of rotary sintering of a more uniform porous structure, and the tribological properties were improved, which is closer to special engineering materials such as PI and PEEK [36]. Meanwhile, it could simplify the conditions and eliminate the pre-pressing process to achieve economical and environmentally friendly effects.

## 2. Experiment

### 2.1. Materials

UHMWPE (XM220, Tm = 143 °C) with a particle size of about 10–30 μm and a molecular weight of 2 × 10^6^ g·mol^−1^ was purchased from Mitsui Chemicals, Inc. (Tokyo, Japan). Lubricating oil (VACTRA OLL NO.2, Mobil Vectra 2) was purchased from Exxon Mobil (Tianjin) Petroleum Co., Ltd. (Tianjin, China).

### 2.2. Fabrication Process of UHMWPE Porous Specimens

**Figure 1:**
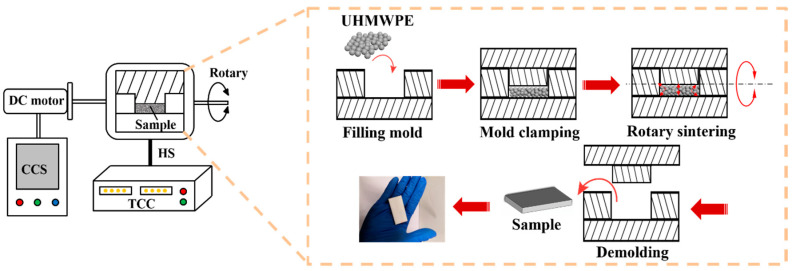
Schematic diagrams of the dynamic rotary sintering device and the fabrication procedure of the ultra-high-molecular-weight polyethylene (UHMWPE) porous materials using the device.

A sintering device, shown in Figure 1, was specially designed and prepared for sintering samples, mainly including a forming mold whose cavity was a regular cuboid shape with sizes of 50 × 25 × 5 mm^3^, an electric heating jacket heating system (including a temperature control cabinet (TCC) and a sensor), and a circuit control system (CCS). During the sintering process, the forming mold was completely covered by the heating sleeve (HS) to achieve a more uniform heating mode. The heating system monitored the temperature of the heating jacket in real-time through a thermocouple. In addition, the circuit control system controlled the motor to realize rotation. When the motor started, the mold rotated 270° back and forth at a speed of 40 r/min under the action of the motor to realize rotary sintering.

Figure 1 shows an illustration of the sintering process. First, the mold was cleaned before the material was filled into the mold, and a layer of release agent was sprayed to facilitate demolding. Then, the UHMWPE powder was loaded into the mold and clamped the mold. Next, the mold was completely covered by an electric heating sleeve and sintered in accordance with the predetermined experimental parameters for heating sintering. In this process, it was divided into static sintering and dynamic sintering. The difference between these two methods was that the mold filled with materials was fixed on a rotating support, and the entire sintering process was completed in stable vibration. When the temperature controller reached the specified sintering temperature and remained stable, it was timed for 10 min. After that, the sintering process was completed and the circuit was turned off. Finally, the sample was rapidly cooled to room temperature with water and then taken out for further analysis.

The interior of the open-type UHMWPE porous material was built up from a series of nearly spherical polymer particles, similar to the most densely packed face-centered cubic structure model in the crystal structure [37,38]. According to the maximum volume utilization of the face-centered cubic model and the volume and logistics density of the mold cavity, the maximum charging amount was 4.0 g, so our initial charging amount started from 4.0 g and decreased gradually.

Considering the effects of sintering time and temperature, the experimental process parameters were determined as shown in Table 1.

### 2.3. Characterization

Macroscopic statistical analysis was used to analyze dimensional stability. The experimental measurement used a Vernier caliper with a measurement accuracy of 0.02 mm. The geometric dimensions of all samples were measured and statistical fitting analyzed, and the size distribution interval was observed to determine the appropriate process conditions and molding methods.

Porosity is an important parameter to characterize the open-type porous material. In this experiment, the porosity of porous materials was indirectly characterized by the oil absorption rate through the vacuum oil absorption experiment. The weight difference ratio of sintered products before and after vacuum oil absorption was used to calculate the oil absorption rate according to Equation (1).

(1) $$\omega =\frac{m_{1}-m_{0}}{m_{0}}\times 100\%$$

where $m_{0}$ is the weight of the sample before oil absorption and $m_{1}$ is the weight of the sample after oil absorption.

The morphology of the surface and cross-section of the samples were observed by an emission scanning electron microscope (QUANTA FEG 250, FEI Company, Hillsboro, Oregon, USA). First, the sintered specimens were immersed in liquid nitrogen and then subjected to brittle fracture. The surface and cross-sections were sputtered with Au in a vacuum, and then examined.

Mechanical properties were evaluated on a universal material testing machine (Instron5566, Shenzhen, China) with the standard of GB/T 104-92, including compressive strength and bending strength. The compression experiment was carried out under the conditions of a compression displacement of 3.0 mm and a compression speed of 1.0 mm/min. As for the bending strength test, the experiment was carried out under the conditions of a span of 40.0 mm and 20.0 mm bending displacement with a speed of 2.0 mm/min. Five samples were tested in each group and averaged. 

The tribological properties of the samples were investigated using ball-disk friction and a wear meter (SFT-2M, Zhongke Kaihua Technology Development Co. Ltd., Lanzhou, China) by sliding them against a GCr15 steel ball of 4 mm in diameter in a circular path of 8 mm in radius for 1 h at a sliding speed of 200 r/min under a normal load of 10 N. The experiment was carried out at room temperature (~22–25 °C) in an atmospheric environment.

The regeneration and long-term stability of the samples are extremely significant in practice. Adsorption/desorption cycles of samples were implemented by a successively high-speed centrifuge and vacuum oil absorption. Under the centrifugal force of 3000 r/min, the oil in the porous structure could be completely removed in 20 min. Then, the sample was re-immersed in the lubricating oil to vacuum-absorb oil and compare the oil absorption rate before and after. The operation was repeated 5 times.

## 3. Results and Discussion

### 3.1. Macroscopic Size of UHMWPE Porous Specimens

**Figure 2:**
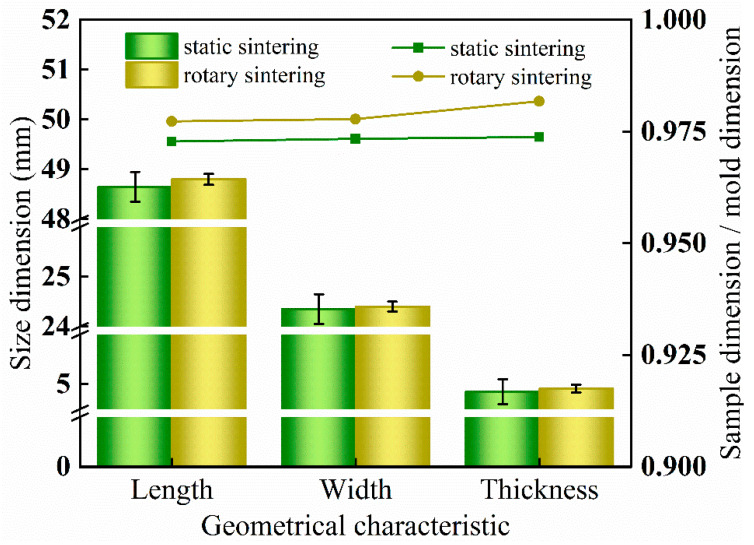
Dimension of samples obtained under the two different molding conditions.

Figure 2 shows the macroscopic size dimension of sintered UHMWPE porous samples. The average sizes of samples obtained by the rotary sintering were higher than the static methods with a smaller deviation. Among the samples of static sintering, the length variation interval was from 48.50 to 48.80 mm, the width was between 24.20 and 24.50 mm, the thickness was between 4.75 and 5.00 mm, and the total range of variation was approximately 0.30 mm, while the size deviation of the samples obtained by dynamic sintering was relatively stable and uniform compared to that static sintered. They were 0.11, 0.10, and 0.08 mm, and the total range of variation was about 0.10 mm. Besides, the ratio between the sample size and the mold cavity size was used to characterize the difference between the actual size of the samples and the theoretical value, among which the ratio of the rotated sintered sample was 0.978–0.982, higher than that of static molding. In static sintering, the sizes of the sample were affected by gravity and uneven heating. The dynamic sintering mold was in a stable mechanical vibration state so that the powder particles were subject to forces from all directions to achieve uniform distribution. Furthermore, this state effectively eliminated the influence of gravity and improved the uniformity of heat transfer, and the dimensional stability of the specimen can be ameliorated at the same time. Thus, it was easy to realize product swap, ensure the durability and continuity of mechanical products, and prolong the service life of products.

### 3.2. Morphology of UHMWPE Porous Specimens

**Figure 3:**
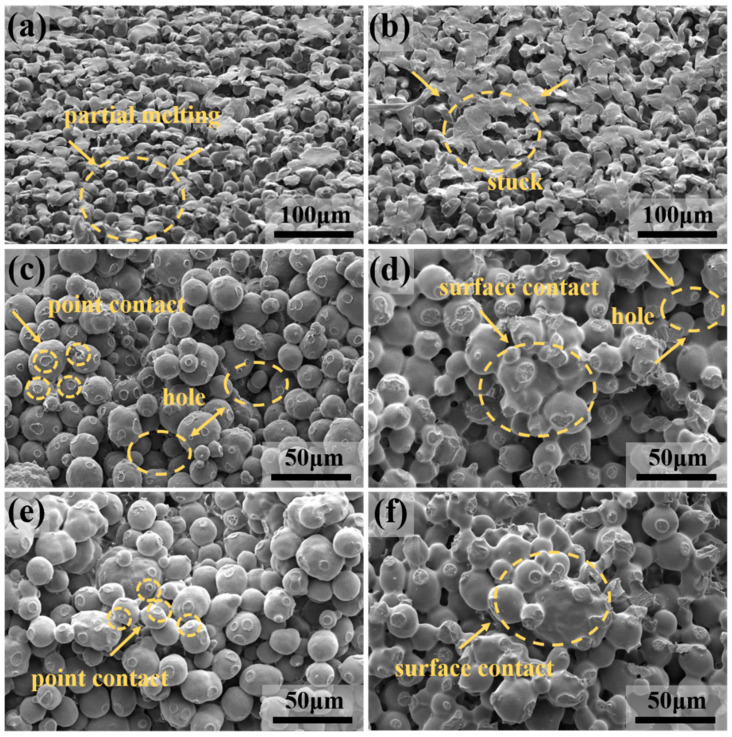
The microstructure of the sample under different sintering temperatures: (**a**,**b**) Surface microstructure of sample under the molding condition of rotary sintering at 170 °C and 200 °C, respectively, where the arrows indicate the melting of samples; (**c**,**d**) cross-sectional microstructure of sample under the molding condition of static sintering at 170 °C and 200 °C, respectively, with the charging amount of 3 g; (**e**,**f**) cross-sectional microstructure of sample under the molding condition of rotary sintering at 170 °C and 200 °C, respectively, with the charging amount of 4 g. Arrows in (**c**–**f**) indicate the melting and the contact mode between the particles and the difference in porous structure of samples obtained by rotary and static sintering methods.

Figure 3 shows the microstructure of the sample under different sintering temperatures and charging amounts. Figure 3a,b compared the melting of particles on the sample surface at different temperatures. The arrow in Figure 3a shows partial melting of the particle surface at a lower temperature (170 °C), while the arrow in Figure 3b shows that at 200 °C, the adjacent particles stuck together and the porosity decreased. Figure 3c–f compares the melting of particles on the sample cross-section between 170 °C and 200 °C. Arrows in Figure 3c,e show that at 170 °C, the particle surfaces were partially melted, similar to point contact between adjacent particles. At the higher temperature of 200 °C in Figure 3d,f, the surface melting of the particles increased, and the adjacent particles were similar to the surface contact. Higher sintering temperatures promoted the heat absorption of the product, leading to a more thorough melting condition; the greater the fluidity of each section, the greater the interpenetration between each section. In addition, the partially molten material filled the gap between the particles, and the porosity of the surface and cross-section of sintered products was reduced. In Figure 3d,f, the arrow in Figure 3d shows uneven stacking of particles; large holes appear, in part, at lower charging amounts (3 g); and in Figure 3f, the particles had a fine-grain arrangement and the porosity decreased. As the charging amount increased, the degree of particle accumulation in dynamic and static sintering increased, the indirect contact of the particles increased, and the probability of occurrence of the larger pores decreased. Therefore, increasing the charging amount was beneficial to controlling the pore size distribution of the porous structure and preparing a porous material with good performance.

**Figure 4:**
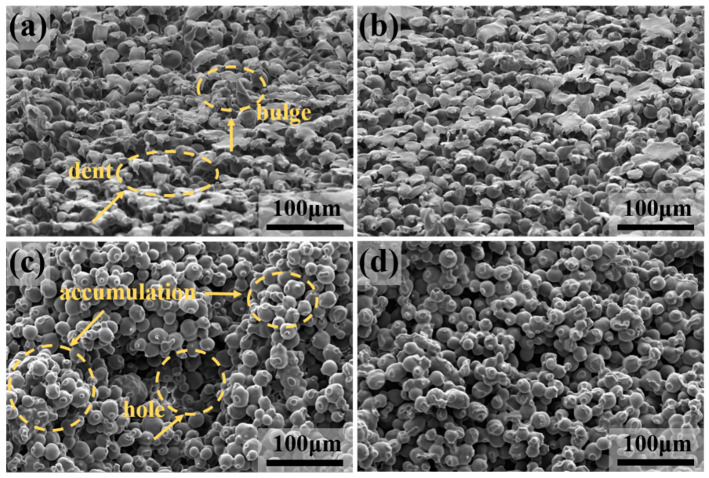
Microstructure of the sample under different forming states: (**a**,**b**) Surface microstructure of the sample under the molding condition of static sintering and rotary sintering, respectively. Arrows in (**c**) indicate the bulges and dents in the surface; (**c**,**d**) cross-sectional microstructure of sample under the molding condition of static sintering and rotary sintering. Arrows in (**c**) indicate the closely packed and large holes of the porous microstructure.

Figure 4 compares different molding states on the surface and cross-sectional microstructure of the samples. In Figure 4a,b, one can see that the micro-surface of the specimen obtained by dynamic sintering was smooth, while the morphology of static sintering was uneven. In Figure 4c,d, the internal structure of the static sintered samples also had significant differences. Some areas were closely accumulated, while in some areas, there were large holes, which were adverse for the samples to maintain uniform and stable performance. Due to the influence of their gravity factors and the difference in the filling process, there were particles partially packed or large holes in the static sintering process. By contrast, dynamic molding effectively avoided this influence by supplying a stable mechanical vibration to the mold in the process of sintering, which caused the powder particles to oscillate from all directions, leading to a more even dispersion structure inside the product and a more regular arrangement of particles. Thus, dynamic molding significantly improved the uniformity of the internal structure of the product and ensured uniformity and stability of the product in actual use.

### 3.3. Porosity of UHMWPE Porous Specimens

**Figure 5:**
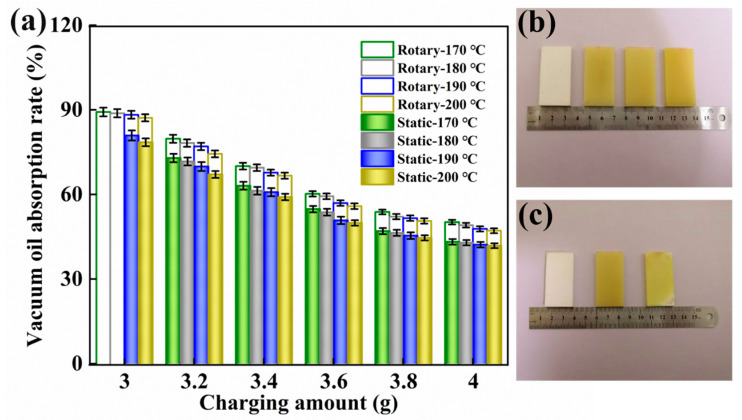
(**a**) The oil absorption rate of the specimen under different process conditions. (**b**) The color change of different samples reflects the amount of oil absorption, where the one on the left is the control group, and the oil absorption rate of samples increases from left to right. (**c**) Comparing the oil absorption rate of the samples obtained by different molding methods, where the left side is the control group, the middle is the rotary sintered sample, and the right is the static sintered sample.

Figure 5 shows the oil absorption rate of sintered products under different sintering process parameters. It can be seen that as the sintering temperature increased, the oil absorption rate of the product gradually decreased, reflecting that the porosity of the products gradually decreased. As the sintering temperature was closely related to the molten state of the particles, the heat absorbed by the powder particles increased as the sintering temperature increased, the chain movement ability was enhanced, the long chains of the particle surface were intertwined and entangled, and the gap of some porous structures was filled by molten particles, so the porosity of the products decreased [39].

Additionally, Figure 5 also presents the oil absorption rate of the products obtained at different charging amounts. With the decrease in the charging amount from 4.0 g to 3.0 g, the height of the column gradually increased, and the oil absorption rate of the product increased from 45% to 89%, which indicates that a 25% change in the amount of filling can double the porosity. The color change of the samples in Figure 5b could also clearly reflect the specific gravity of the adsorbed oil. From left to right, the more oil content the sample absorbed, the darker the color of the sample. As the volume of the mold cavity was constant, as the charging amount of UHMWPE powder decreased, the powder particles were loosely accumulated, the gap between the particles increased, and the porosity of the sample increased. 

Different molding states also influenced the porosity of the sintered products. In Figure 5a, the oil absorption rate of the prepared samples was generally higher than that of static sintering under dynamic sintering conditions at different temperatures and charging amounts. Moreover, it can be directly observed in Figure 5c that a few white unabsorbed parts still remained inside the product after the static molded product was sucked, the dynamic molded product had a transparent appearance after oil absorption, and no concentrated agglomeration occurred. It was fully proved that the stable mechanical vibration promoted the uniform distribution of materials and improved the effectiveness of heat transfer, which reduced the accumulation and agglomeration of materials to some extent.

### 3.4. Mechanical Properties of UHMWPE Porous Specimens

**Figure 6:**
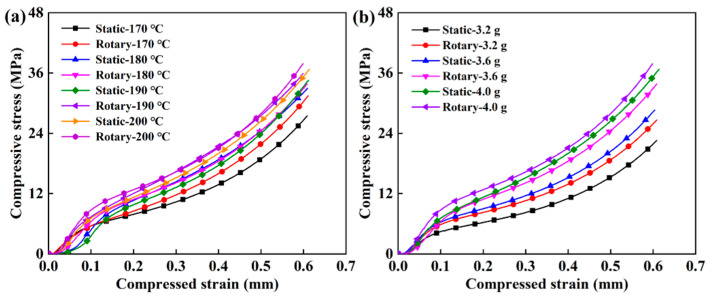
The compressive strength of the sample under different process conditions: (**a**) Trends of compression properties of samples obtained by different molding methods with respect to the change in temperature when the charging amount was 4 g, and (**b**) trends of compression properties of samples obtained by different molding methods with respect to the change in charging amount when the sintering temperate was 200 °C.

**Figure 7:**
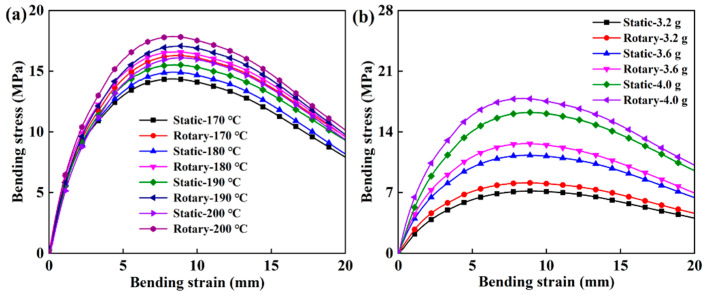
The bending strength of the product under different process conditions: (**a**) Trends of bending behavior of samples obtained by different molding methods with respect to the change in temperature when the charging amount was 4 g, and (**b**) trends of bending behavior of samples obtained by different molding methods with respect to the change in charging amount when the sintering temperature was 200 °C.

Figure 6 and Figure 7 show a comparative analysis of the compressive strength and bending strength of the samples obtained in different sintering processes. As the sintering temperature increased, the compressive strength and bending strength of the obtained sample gradually increased, and the change in charging amount had a greater influence on the mechanical properties. With the increase in temperature and the influence of polymer particle crystallization and long-chain inter-diffusion, the interfacial bonding strength of the sample and, correspondingly, the mechanical strength improved [40].

The compressive strength of the sintered samples with different process parameters is shown in Table S1. The compressive strengths of the obtained samples were between 20 and 38 MPa. It can be confirmed that the pore distribution of the porous structure was reasonable from the mechanical properties. It was also closely related to the particle size of the material. The original constituent particles of XM220 powder were uniform particles with a particle size of 10–30 μm. The size of the particles was fine and uniform, which facilitated contact between the particles. Therefore, the obtained porous structure was more compact and had strong mechanical strength. With the increase in temperature, the compressive strength of the sample prepared by dynamic molding was steadily increasing and tended to increase linearly, while the static molded sample had an overall increase in compressive strength. However, there was a special case; as shown in Table S1, the individual compressive strength decreased from 26.63 to 25.46 MPa, which was related to gravity effects and uneven charging. Figure 6 shows the compressive stress–strain curve of different samples, characterizing the compressive capacity of the samples at 60% thickness. As a porous material, the sample first underwent a compaction stage and the modulus was relatively small; then, it was equivalent to the compression of the compacted sample, the modulus became larger, and, after the yield point, the curve continued to rise until the end of the experiment. Figure 6a shows the compressive strength of samples with different sintering temperatures and sintering conditions at 4 g. When the temperature rose and the porosity decreased, the porous structure was compact and the ability to resist external axial stress increased, so the compressive strength correspondingly increased. The effect of the charging amount on the compressive strength of the sample was similar to the temperature and was more pronounced than temperature. As shown in Figure 6b, as the charging amount increased from 3.2 g to 4 g, the compressive strength of the sample increased about 11 MPa, and the compression strength of the samples improved uniformly by dynamic molding.

The bending strength can comprehensively reflect the bearing capacity of the porous structure. In Table S2, the obtained samples had a wide range of bending strengths between 6 and 18 MPa. As the temperature increased from 170 °C to 200 °C, the bending strength of the dynamically sintered samples increased linearly and was higher than that of static sintering. It further demonstrated that dynamic sintering was more conducive to obtaining a more stable and uniform porous structure and improving the mechanical strength of the sample. As revealed in Figure 7a, the bending stress increased as the porosity was lowered, while the temperature rose and the porous structure was more compact. In Figure 7b, as the mass of the charging amount increased, the bending stress had the same trend as the change in temperature. An additional amount of 0.8 g could produce a bending strength change of about 10 MPa. The overall performance of the dynamically sintered sample was better than that of the static sintered sample.

Thus, the compressive strength and bending strength of the samples obtained by dynamic sintering were improved compared to static sintering. The dynamic sintering process mold had been in a stable state of vibration, and the probability of contact between each particle and the size of similar particles through the contact between the formations of the gap was even more uniform, so the internal structure of the product was more uniform and the mechanical properties enhanced. 

### 3.5. Friction and Wear Performances of UHMWPE Porous Specimens

**Figure 8:**
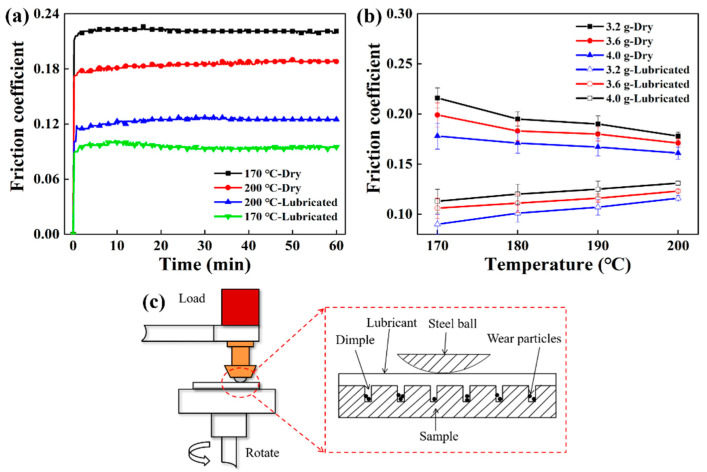
The friction and wear performance comparison of the samples: (**a**) The friction curve of the sample under different porosity and working conditions when the charging amount was 3.2 g, (**b**) coefficient of friction of samples under different porosity and working conditions, and (**c**) schematic diagram of friction and wear under different working conditions.

The previous experiments had shown that dynamic rotary sintering can obtain a uniform porous structure. The following article discussed the tribological properties of the obtained samples and verified their performance in practical applications. Figure 8a illustrates the friction coefficients of the samples under dry friction and oil lubrication conditions. Among them, the friction curve of samples quickly reached the peak and stayed in a steady state. Figure 8b shows that the coefficient of friction of the samples increased from 0.17 to 0.22 as the porosity increased in dry friction, while it decreased from 0.13 to 0.08 as the porosity increased in the oil lubrication condition. Figure 8c displays the changes in the surface of the sample during friction and wear. At dry friction, as the porosity increased, the surface porosity of the sample was larger, and the more pores on the surface increased the surface roughness, so the friction coefficient of the UHMWPE porous materials reached up to 0.22 [41]. Under oily conditions, changes in the porosity of the specimen resulted in changes in surface roughness and in the storage and output of the porous sample. At the beginning of the friction test, the oil stored in the porous sample was squeezed to the surface of the friction pair to remove some of the heat and lubrication. At this point, for the porous UHMWPE sample, the effect of oil on the friction coefficient was dominant, while the effect of surface roughness was relatively small. Therefore, the friction coefficient of the sample can reach as low as 0.08, reducing by about 56%.

For UHMWPE porous samples of the same porosity, the coefficient of friction under oil-lubricated conditions was lower than that under dry friction. The possible reason was that under dry friction conditions, due to the continuous action of the contact stress between the friction pairs, the temperature of the friction pair surface increased with the generation of wear debris, which made the friction condition between the friction pairs worse [15]. Under oil-lubricated conditions, the oil stored inside the porous UHMWPE sample was continuously squeezed out, and the oil could take away part of the heat and played a role in secondary lubrication, improving the lubrication condition of the friction pair surface [42,43]. Therefore, the friction coefficient of porous UHMWPE was about 0.1 under oil lubrication. Considering the porosity, mechanical properties, and friction coefficient, the optimal process parameters of the UHMWPE porous material were as follows: The sintering temperature was 180 °C or 190 °C, and the charging amount was between 3.6 g and 3.8 g.

**Figure 9:**
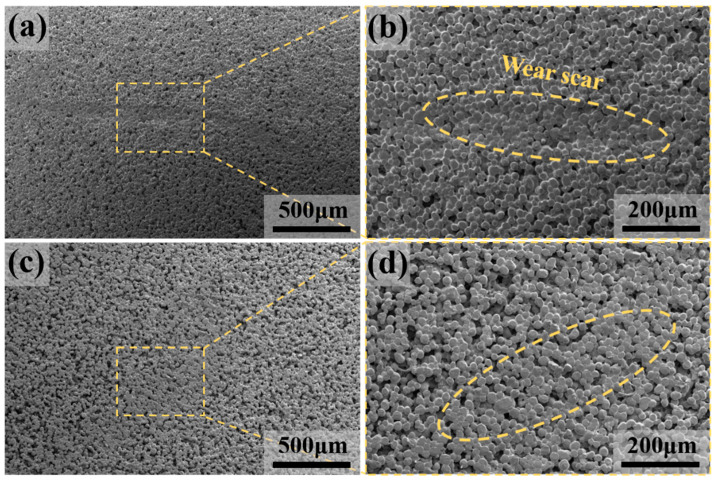
The SEM image of wear scar under dry friction, and (**b**) wear scar diagram in the yellow circle of (**a**) at higher magnification; (**c**) the SEM image of wear scar under oil lubrication conditions, and (**d**) wear scar diagram in the yellow circle of (**c**) at higher magnification.

The surface morphologies of the wear scar obtained by the friction and wear test are displayed in Figure 9. In the early stage of the experiment, the oil adsorbed by the samples was removed with cyclohexane and the samples were then dried at 80 °C to volatilize the cyclohexane; then, they were subjected to brittle fracture, where the surface microtopography was observed. Figure 9a,b show an obvious wear scar. In the case of no oil lubrication, the friction between the pairs was exposed, and the porous structure was subjected to large compressive stress, resulting in a more obvious wear scar. However, the porous structure was not subject to significant damage and maintained a relatively complete microscopic morphology, which was closely related to the excellent mechanical properties of UHMWPE. In Figure 9c,d, the porous structure was squeezed to a certain extent, but there were no obvious scratches. This was because the lubricating oil formed a continuous phase between the friction pairs, avoiding the direct contact between the two, and acted as secondary lubrication, thereby improving the lubrication condition of the friction pair surface and reducing the wear rate [44]. 

**Figure 10:**
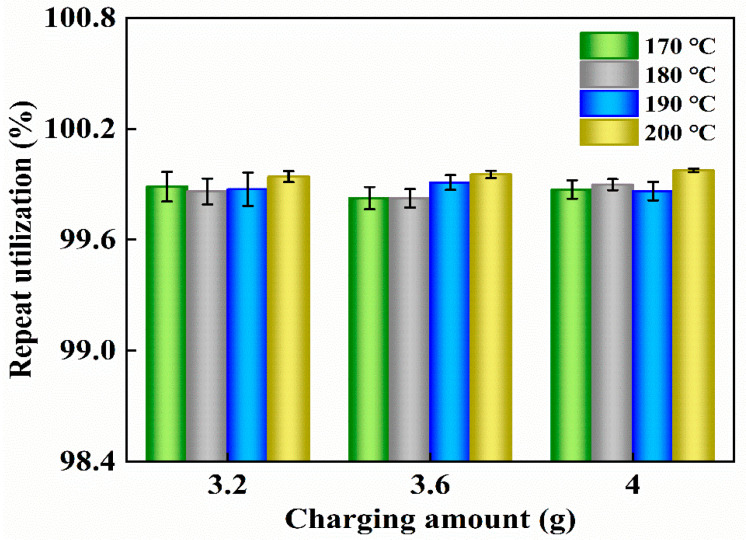
The repeat utilization of the product under different process conditions.

The open-type UHMWPE porous material passed through the connected porous structure to save oil. Under the condition of no external force at room temperature, the oil content of the sample did not change substantially in the observation time of one year and had a good oil retention effect. The repeated use of the obtained sample is shown in Figure 10. It can be found that the sample repeat utilization was as high as 99%, which can achieve the purpose of recycling, and was beneficial to resource reuse and economic benefit.

## 4. Conclusions

In this paper, UHMWPE porous self-lubricating materials had been successfully fabricated via a rotary sintering technique. The mold in the entire sintering process was in a stable state of mechanical vibration, which can effectively improve the loading process caused by the phenomenon of local material density accumulation and improve the material in all directions of the movement probability, increasing the material contact point. Under the same sintering process parameters, the dynamic molding can be used to prepare the UHMWPE porous materials with good stability, uniform porosity, excellent mechanical properties, and high forming efficiency. The following optimum sintering conditions were shown: Sintering temperature of 180 °C or 190 °C, sintering time determined as 10 min, and loading capacity of between 3.6 g and 3.8 g. The friction and wear experiments show that the obtained porous material had a relatively stable and small friction coefficient, and the friction curve was relatively stable. Moreover, the friction coefficient of porous UHMWPE under oil lubrication was about 0.1 and the wear rate was lowered. It can be used in oil-containing self-lubricating materials and had a recycling rate of up to 99%, which is in line with the requirements of recycling production. Therefore, this method provides a new idea for the preparation of porous self-lubrication materials for robot joints and drones applications.

## Figures and Tables

**Table 1:** The sintering conditions of the examined samples.

Sintering Method	Time (min)	Temperature (°C)	Charging Amount (g)
static	10	170, 180, 190, 200	3, 3.2, 3.4, 3.6, 3.8, 4.0
dynamic	10	170, 180, 190, 200	3, 3.2, 3.4, 3.6, 3.8, 4.0

## Supplementary Materials

The following are available online at https://www.mdpi.com/2073-4360/12/6/1335/s1, Table S1: The compressive strength (MPa) of the samples under different process conditions, Table S2: The bending strength (MPa) of the samples under different process conditions.
