# Supplementary material for: Fabrication of Self-Lubricating Porous UHMWPE with Excellent Mechanical Properties and Friction Performance via Rotary Sintering
Source: Polymers (Basel). 2020 Jun 12;12(6):1335. doi: 10.3390/polym12061335 (PMC7361689; doi:10.3390/polym12061335)
Supplement: Supplementary file 1 [file polymers-12-01335-s001.pdf]

Table S1 The compressive strength (MPa) of the samples under different process conditions.

Temperature (°C)	Molding state	Charging amount（g）					
		3.0	3.2	3.4	3.6	3.8	4.0
170	static	--	19.79	21.31	26.63	27.34	27.83
	dynamic	19.99	22.80	24.29	29.51	30.67	31.83
180	static	--	21.68	22.25	25.46	29.64	32.99
	dynamic	21.88	24.20	26.67	30.70	32.58	33.83
190	static	18.47	20.26	23.63	24.16	31.27	34.57
	dynamic	22.74	25.00	27.68	32.51	34.73	36.09
200	static	21.76	22.62	27.51	28.46	34.12	36.74
	dynamic	25.05	26.35	31.21	33.67	36.83	37.89

Table S2 The bending strength (MPa) of the samples under different process conditions.

Temperature (°C)	Molding state	Charging amount（g）					
		3.0	3.2	3.4	3.6	3.8	4.0
170	static	--	5.80	7.56	10.56	12.65	14.32
	dynamic	6.03	7.46	9.24	11.58	13.78	16.29
180	static	--	6.70	8.66	10.88	13.06	14.81
	dynamic	6.14	7.69	9.55	11.88	13.97	16.43
190	static	5.28	6.83	9.01	11.26	13.44	15.36
	dynamic	6.27	7.78	9.86	12.05	14.30	16.85
200	static	5.62	7.25	9.31	11.32	13.42	16.25
	dynamic	7.13	8.13	10.27	12.65	14.81	17.85
